# Slaughter Conditions and Slaughtering of Pregnant Cows in Southeast Nigeria: Implications to Meat Quality, Food Safety and Security

**DOI:** 10.3390/foods10061298

**Published:** 2021-06-05

**Authors:** Ugochinyere J. Njoga, Emmanuel O. Njoga, Obichukwu C. Nwobi, Festus O. Abonyi, Henry O. Edeh, Festus E. Ajibo, Nichodemus Azor, Abubakar Bello, Anjani K. Upadhyay, Charles Odilichukwu R. Okpala, Małgorzata Korzeniowska, Raquel P. F. Guiné

**Affiliations:** 1 Department of Veterinary Obstetrics and Reproductive Diseases, Faculty of Veterinary Medicine, University of Nigeria, Nsukka 410001, Nigeria; ugochinyere.njoga@unn.edu.ng; 2 Department of Veterinary Public Health and Preventive Medicine, Faculty of Veterinary Medicine, University of Nigeria, Nsukka 410001, Nigeria; obichukwu.nwobi@unn.edu.ng; 3 Department of Animal Health and Production, Faculty of Veterinary Medicine, University of Nigeria, Nsukka 410001, Nigeria; festus.abonyi@unn.edu.ng; 4 Department of Animal Science, Faculty of Agriculture, University of Nigeria, Nsukka 410001, Nigeria; henry.edeh@unn.edu.ng; 5 Department of Animal Health and Production, Enugu State Polytechnic, Iwollo 401139, Nigeria; ejikeajibo5@gmail.com (F.E.A.); azornichodemus@gmail.com (N.A.); 6 Faculty of Veterinary Medicine, Wroclaw University of Environmental and Life Sciences, 50-375 Wrocław, Poland; 7 Heredity Healthcare & Lifesciences, 206-KIIT TBI, Patia, Bhubaneswar, Odisha 751024, India; upadhyayanjanikumar6@gmail.com; 8 Faculty of Biotechnology and Food Sciences, Wroclaw University of Environmental and Life Sciences, 50-375 Wroclaw, Poland; malgorzata.korzeniowska@upwr.edu.pl; 9 CERNAS Research Centre, Polytechnic Institute of Viseu, 3504-510 Viseu, Portugal

**Keywords:** animal welfare, bovine foetal wastage, food safety and security, meat quality, national herd size, slaughter of pregnant cows

## Abstract

The increase in the slaughter of pregnant cows (SPCs) for meat (except as may be approved by veterinarians on health grounds to salvage the animal) is unethical. SPCs for meat is also counterproductive, detrimental to food security, and may enhance zoonotic disease transmission. In this context, therefore, this current study examined slaughter conditions and the slaughtering of pregnant cows, and the implications for meat quality, food safety, and food security in Southeast Nigeria. The direct observational method was employed to examine the slaughterhouse activities, from when the cattle arrived at the lairage to the post-slaughter stage. A pre-tested and validated closed-ended-questionnaire was used to elicit information on causes of the SPCs and the method of disposal of eviscerated foetuses. Pregnancy status of cows slaughtered was determined by palpation followed by visual examination of the eviscerated and longitudinal incised uteri. The study lasted for six months during which 851 cows out of 1931 slaughtered cattle were surveyed. Assessment/decision-making protocol of slaughterhouse conditions, welfare conditions of slaughter-cattle, reasons for sale or slaughter of pregnant cows, distribution of pregnant cows slaughtered, method of disposal of eviscerated foetuses, and estimated economic losses of SPCs were delineated. Of the 851 cows examined, 17.4% (148/851) were pregnant while 43.2% (64/148) of the total foetuses recovered were in their third trimester. Major reasons adduced for SPCs by proportion of involved respondents were: ignorance of the animals’ pregnancy status (69.7%, 83/119), high demand for beef (61.3%, 73/119), preference for large-sized cattle (47.9%, 57/119), economic hardship (52.1%, 62/119) and diseases conditions (42.9%. 51/119). The conduct of SPCs for meat would not be profitable. This is because within six months, an estimated loss of about 44,000 kg of beef, equivalent to ₦ 70.1 million or $186,400 would be associated with SPCs and the consequential foetal wastages. If losses were to be replicated nationwide across slaughterhouses, 4.3 tons of beef estimated at ₦ 8.6 billion or $23 million would be wasted. Improving slaughter conditions and the welfare of slaughter-cattle in Nigerian slaughterhouses through advocacy, training of slaughterhouse workers, and strict implementation of laws promoting humane slaughter practices is imperative. Preventing SPCs for meat and inhumane slaughter practices at the slaughterhouse would enhance the welfare needs of slaughter cattle, grow the national herd size, and improve meat safety as well as food security.

## 1. Introduction

Globally, achieving high-level beef/meat quality from any given cattle slaughterhouse requires that optimum levels of good practices must be upheld. Regardless of location, cattle pre-slaughter and slaughter operations remain very critical to meat quality and food security [1,2,3]. There are pre- and post-slaughter conditions/factors in slaughterhouses that influence meat quality and food security. For instance, pre-slaughter factors include high animal density during transport, inadequate lairage facilities at the slaughterhouse, long-distance travel, (poor) handling practices during loading and unloading and during transport, as well as unskilled drivers [4,5,6,7]. On the other hand, post-slaughter factors include humidity, temperature, meat pH, microbial contamination and storage time, which would affect the keeping and nutritional qualities of the meat [8]. Moreover, the welfare of animals at slaughter indeed affects meat quality, which undermines food safety, public health, and economic viability in meat production or processing enterprises [2,9]. Additionally, the stress inflicted during slaughter can change the meat characteristics [10,11,12]. Further, bad slaughter management operations can decrease carcass quality, which could arise because of cross-contamination [2,13]. Some carcass/meat defects caused by pre-slaughter stress or poor welfare conditions are Pale, Soft, Exudative (PSE) and Dark Firm and Dry (DFD) meats [14,15]. Humane handling of slaughter-cattle prohibits cruel practices such as dragging, dropping, throwing or hoisting during the slaughter process [16]. Factors that are used to evaluate compliance to humane slaughter practices include the proportion of the slaughter animals that: (a) were successfully stunned at the first attempt; (b) were rendered unconscious post first stunning; (c) vocalised during stunning and bleeding; (d) fall during handling; and (e) moved with an electric goad [16,17].

Animal welfare (AW) refers to the overall well-being of non-human animals, especially domesticated/farmed animals reared for companionship and food (meat) production [18]. Typically, an animal is considered to be in a good state of welfare when it is healthy and well-nourished, under zero pain, distress or fear, and able to easily express its instinctive behaviour [18]. Good AW, among other things, requires the provision of appropriate feed and shelter, humane handling, and humane slaughter [19]. The principles of AW permit responsible use of animals for human benefits (companionship, food, recreation and work), but such animals must be protected and cared for in a manner that minimises fear, pain, stress, and suffering [20]. In Nigeria, the Department of Veterinary and Pest Control Services (DVPCS) is responsible for protection and promotion of AW. Actually, the DVPCS in Nigeria is under the supervision of the Chief Veterinary Officer (CVO), who also functions as the World Organisation for Animal Health delegate. The CVO oversees animal health and welfare issues, well known to involve: (a) animal disease prevention and control; (b) prevention of the slaughter of gravid animals (unless approved by an accredited veterinary doctor); and (c) humane animal handling/slaughter practices.

Adherence to AW standards, other than an ethical issue, may enhance the microbiological safety of beef and beef products, and therefore protect the consumers from infections with zoonotic pathogens. Stress factors and poor AW can increase susceptibility of slaughter-cattle, especially the pregnant ones, to diseases which may be zoonotic and hence transmissible to humans via the food chain [21]. Globally, approximately 600 million foodborne illnesses and 420,000 deaths have annually been attributed to microbial pathogens, largely owed to poor food (as well as meat) processing practices [9]. Through the provisions of the Meat Edict of 1968 in Nigeria, the slaughter of pregnant female animals (PFAs) is prohibited, so as to improve AW and conserve livestock resources. This specific edict equally forbids the slaughter of gravid animals, with the exception of emergency slaughter. The purpose for this is to relieve animal suffering, and this has to be recommended by a veterinarian. Despite the obsolete nature of this edict, maternal slaughter and the resultant foetal wastage has continued unabated [22,23,24]. The reason for this could also be attributed to the poor implementation of this edict. Moreover, the immediate cause(s) of this menace (maternal slaughter) still remains elusive. This may be due to the upsurge in the demand for edible animal products in Nigeria [25], occasioned by the rapidly increasing human population, currently estimated at 210 million, which is based on a 3.5% annual growth rate [26].

Besides animal cruelty, the slaughter of pregnant female animals (PFAs) remains counterproductive, threatens food security, and exerts immense loss in livestock revenue/resources [27,28,29,30]. Slaughter of pregnant cows (SPCs) would not only depopulate productive female animals, but also depletes future herds via consequential foetal wastages. As such, this would jeopardise efforts towards achieving self-sufficiency in provision of enough edible animal protein, especially in developing countries [31]. SPCs could lead to the introduction of exotic zoonoses (bovine spongiform encephalopathy and variant Creutzfeldt–Jakob disease) through meat importation due to deficits in animal protein occasioned by excessive off-takes from the national herd [31]. In developing countries, the diminution of animal protein is a major public health problem associated with the slaughter of PFAs [32], which may ensue through the unwarranted off-takes from the national herd without commensurate replacement, as epitomised in SPCs and the consequential bovine foetal wastages. Despite the fact that SPCs and associated AW and economic implications are preventable, the practice regrettably persists in Nigeria [24,33,34,35,36,37], where demand for edible animal products incidentally supersedes the supply [38,39].

SPCs appears to increasingly be the status quo, despite the fact that unauthorised slaughter of gravid females for meat connotes animal cruelty and counteracts the growth of the national herd. Therefore, determining the current status and its underpinning drivers are warranted considering that the last available report on SPCs in South-East Nigeria is over three decades old [40]. Additionally, it appears there is no published report/pathway to determine the conditions/welfare of both slaughtered cattle/slaughterhouses in Nigeria. Additionally, knowing the number of pregnant cattle slaughtered for meat may provide useful clues on slaughterhouses’ compliance to AW needs, especially in Nigeria. Further, determining the root-causes of SPCs will guide informed decision making regarding improved AW, growing the national cattle herd size and boosting the livestock economy in Nigeria. To supplement existing literature, therefore, this current study examined the slaughter conditions and slaughtering of pregnant cows, and the implications for meat quality, food safety, and food security in South-East Nigeria. 

## 2. Materials and Methods

### 2.1. Area/Location of Study

**Figure 1:**
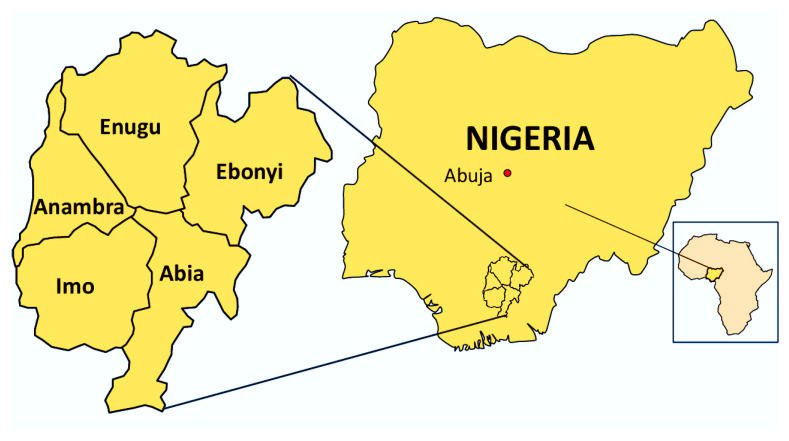
Map of the study area, the South-East geopolitical zone of Nigeria, showing the positions of the zone in African and Nigerian maps, and the constituent five Nigerian states that comprise the South-Eastern part.

The demographics, climatic and orographic conditions of South-East Nigeria, the study location, have been previously described [41,42]. The South-East is one of the six geopolitical zones of Nigeria that consists of five states—Abia, Anambra, Ebonyi, Enugu and Imo (Figure 1). The zone is located on latitude 5°45′00′′ N and longitude 8°30′00′′ E. The South-East zone has a total land area of about 40,000 km^2^, and borders Cameroon at the Eastern boundary. The region had an estimated population of about 40 million people in 2019 [43], but realistically the population is now approximately 60 million. The population density of the South-East ranges from 140 to 390 persons per km^2^ (depending on the state or town), as against Nigeria’s average population density of 226 per km^2^; making the zone one of the most densely populated regions in Africa.

### 2.2. Schematic Overview of the Experimental Program

**Figure 2:**
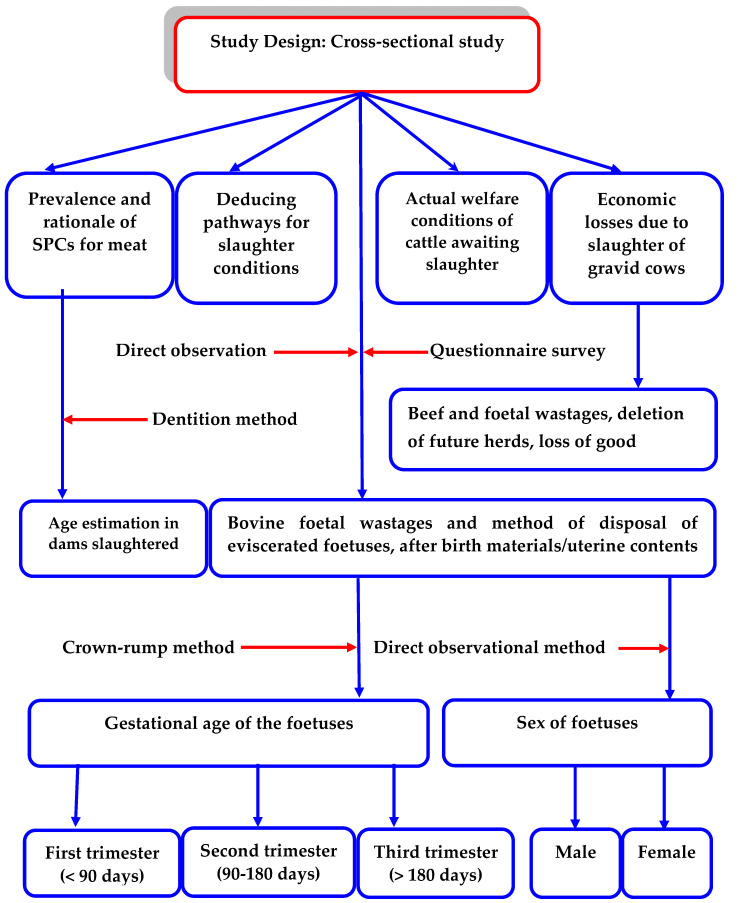
The schematic overview of the experimental program, from the study design, to the age estimations, and the disposal of eviscerated bovine foetuses from major slaughter facilities in South-East Nigeria.

The schematic overview of the experimental program, from the study design to the age estimations and disposal of eviscerated bovine foetuses from major slaughter facilities in South-East Nigeria, is shown in Figure 2. For emphasis, this current study was designed to examine how slaughter conditions and slaughtering of pregnant cows impact on the meat quality, food safety, and food security, with specific reference to the South-Eastern part of Nigeria. The study adopted a cross-sectional study design which consisted of two phases. The first was the pathway determination of the status of slaughter-cattle, from the process of off-loading, to the period of waiting at the lairage, and during slaughter. This was conducted by direct observational methods, as described by Holmes [44]. The second was determination of the prevalence and causes of SPCs in the study area. Three major slaughter facilities in the study area, Nsukka, Kwata and Akwata slaughterhouses, being the major slaughterhouses with a combined slaughter capacity of about 70% of all cattle consumed in the zone [45], were purposively selected for the study.

### 2.3. Ethical Approval

Institutional ethics approval was deemed not necessary for this survey activity. This is because the researchers did not slaughter the cattle surveyed, but only examined slaughtered pregnant cows during routine meat/carcass inspection activities performed at the studied slaughterhouses. Pre-slaughter conditions of the animals were determined by direct observational study, and there was no physical restraint/handling of the live animals by the researchers. However, the slaughterhouses’ associations approved the use of the research instrument (i.e., questionnaire) prior to the survey. Importantly, given that the survey involved interviews of slaughterhouse workers, informed consent was orally obtained prior to their participation. Additionally, this study adhered to the code of ethics of the World Medical Association Declaration of Helsinki [46], and the participation of every respondent included in this survey was entirely voluntary.

### 2.4. Research Visits and Sample Size Determination

The selected slaughterhouses were visited weekly, precisely on Saturdays (as more cattle are usually slaughtered on weekends) for six months, (three months during the dry/hot season and another three months during the wet/rainy season) for data collection. A minimum sample size of 385 cows was calculated for the study on pregnancy status of slaughter-cattle, using the formula: *n* = Z^2^ P (1 − P)/d^2^; where *n* = required sample size, Z = normal deviate (1.96) at 5% significance level, and P = assumed prevalence of bovine foetal wastages (BFW). The sample size calculation was based on a 50% estimated prevalence, as described by Pourhoseingholi and other workers [47], since there is no published report, to the best of knowledge, on BFW in South-East Nigeria. However, for accuracy and buoyancy of data, a total of 851 cows out of 1931 slaughtered cattle surveyed during the six month period, were selected by simple random sampling technique (toss of coin) in this study.

### 2.5. Development and Validation of Research Instrument

The research instrument took the form of a structured and pre-tested closed-ended questionnaire. This was used to elicit information underscoring the SPCs and its implications on the meat quality, food safety and security. Specifically, the questionnaire structure had two parts. One part sought to ascertain the pathways to determine the slaughter conditions/welfare. This involved questions that aimed to reveal the major steps followed prior to decision-making about welfare conditions within the studied slaughterhouses. The other part sought to ascertain the possible causes of SPCs and method of disposal of eviscerated foetuses or gravid uterine contents, from which implications of SPCs on the meat quality, food safety and security could be deciphered. This involved questions that aimed to reveal the reasons for sale or slaughter of pregnant cows, distribution of pregnant cows slaughtered, the method of disposal of eviscerated foetuses, and the estimated economic losses of SPCs.

The initial draft questionnaire was subjected to face and content validations consistent with the method described by Bolarinwa [48]. Thereafter, it was pilot tested on 20 respondents in order to detect and correct possible errors that may arise during the actual survey process. Additionally, Cronbach’s alpha test was performed to ascertain the reliability of the data/questionnaire in determining the parameters of interest, using IBM^®^ SPSS statistics version 25 (SPSS Inc., Chicago, IL, USA). The test yielded a reliability coefficient (alpha value) of 0.841 (which was ≥ 0.7), and therefore ascertained the questionnaire reliable and valid, and ready for this current work.

### 2.6. Administration of Research Instrument

A total of 119 slaughterhouse workers, selected from a sampling frame of 356 workers, across the three selected slaughterhouses, participated in the study. The slaughterhouse workers surveyed included butchers, cattle dealers/marketers, animal attendants (herders) and meat sellers. These workers performed various roles, one complementing the other in some cases. For instance, a butcher could be herding the cattle after abattoir operations. In another instance, the cattle marketers may butcher the slaughtered animals and also sell the meat themselves. A simple random sampling technique (toss of coin) was used to select the 119 respondent from over 200 slaughterhouse workers, who willingly volunteered to partake in the survey.

The questionnaire survey was conducted during each visit. The survey was conducted in the form of an interview, and the responses recorded accordingly. The veterinarian and an assistant would ask questions from the questionnaire, and the slaughterhouse worker would provide answers. In some cases where respondents were unable to fully understand the question posed in the English language, the native language was used without change of content or context, but to enhance participation, ensure least pressure (to the respondent), and elicit appropriate response. Utmost care was taken to ensure that each respondent was not surveyed more than once.

### 2.7. Elaboration of Questionnaire Parameters

#### 2.7.1. Pathways to Determine the Slaughter Conditions

The task here was to determine what would eventually be called “assessment/decision-making protocol of slaughterhouse conditions/welfare”. This would help identify and subsequently establish in logical sequence the steps that would lead to decision-making as regards conditions/welfare of slaughterhouses. This involved questions aimed to delineate major factors that could serve as checks, as well as help establish right decisions regards the condition/welfare of the slaughterhouse. Brainstorming sessions with (slaughterhouse) workers, together with the veterinarians that conducted the study, would enable deductions/reflections on the positive and negative welfare conditions, to proffer potential way-outs.

#### 2.7.2. Pregnancy Status of the Selected Slaughtered Cows

The pregnancy status of the selected slaughtered cows was ascertained by visual inspection and palpation of the uteri and the uterine horns for gross/macroscopic evidence(s) of pregnancy. The uteri and the horns were eviscerated post slaughter, longitudinally incised, and then systematically and thoroughly inspected (post-mortem) for evidence of pregnancy (presence of membrane slip, foetus/foetuses, placentation, placentome-foetal cotyledon and maternal caruncle or other afterbirth materials). The ages of the dams were estimated by the dentition method as described by Pace and Wakeman [49]. For the foetuses, estimation of the gestational age was performed by crown-rump length measurement using the formula: X = 2.5 (Y + 21); where X = age of the foetus in days and Y = crown-rump length in cm as described by Kouamo and other researchers [50]. Thereafter, the gestational age of the recovered foetuses were categorised as first (<90 days), second (90–180 days) or third (>180 days) trimester. Similarly, sex of the foetuses was determined by visual examination of the external genitalia at the inguinal region or below the base of the tail.

#### 2.7.3. Estimated Loses of Beef from Slaughtered Pregnant Cows (SPCs)

Losses in beef production associated with SPCs was estimated based on a 63% carcass yield [51], and an average maturity live weight of 460 kg per cattle, as described by Ndi and co-workers [52]. The estimation assumed that the 148 foetuses would be born alive and raised to maturity, but allowance for 5% pre-maturity mortality was factored in as recommended by Ndi and co-workers [52]. Additionally, the associated economic losses were determined by mark-up pricing methods, as described by Crowson [53], using the prevailing price of beef in the local markets. The monetary value was estimated in Naira (₦) and converted to US Dollars ($) based on the current market price of ~₦ 2000 (roughly $5.3 at the Central Bank of Nigeria (CBN) official exchange rate of ₦ 380 per US Dollar as of 01 April 2021) per kilo of beef.

### 2.8. Statistical Analysis

The resultant data were collated, analysed and presented in tables and figures. Fisher’s exact test was used to determine whether there is significant association (*p* ≤ 0.05) between SPCs and seasons, age, breed, months of the year and slaughterhouse locations. The statistical significance was set at *p* < 0,05 (95% confidence interval). IBM^®^ SPSS statistics version 25 (SPSS Inc., Chicago, IL, USA) was used to run the statistical analysis.

## 3. Results

### 3.1. Establishing the Pathway of Slaughter Conditions/Welfare

**Figure 3:**
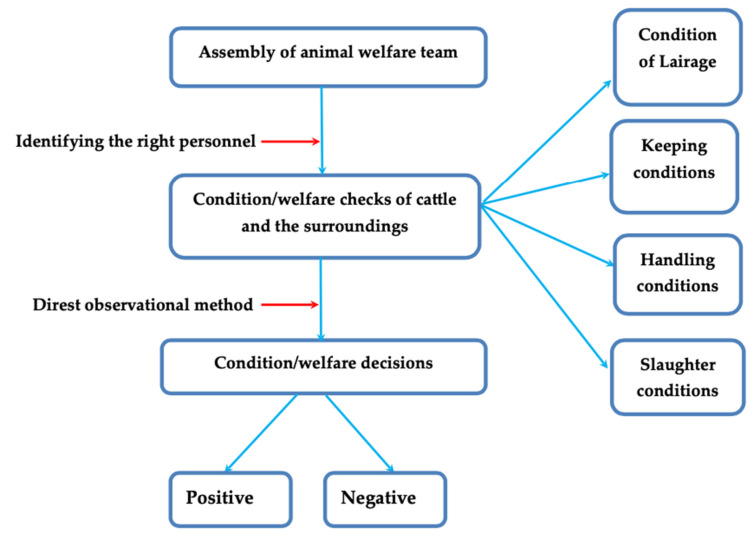
A schematic flow of assessment/decision-making protocol of slaughterhouse conditions/welfare.

Brainstorming sessions with (slaughterhouse) workers, together with the veterinarians that conducted the study, enabled the deduction of the feasible pathway in regard to the slaughterhouse conditions/welfare decision-making activity. The schematic diagram of assessment/decision-making protocols of slaughterhouse conditions/welfare is shown in Figure 3. It shows the major steps, from the assembly of the animal welfare team, to the actual decision-making actions. Table 1 shows the considerations of cattle slaughter conditions/welfare and corresponding way-outs, from environment at the lairage, to during the slaughter of cattle. The considerations of cattle slaughter conditions/welfare were either positive or negative. The way-outs were aimed to alleviate the negative (and strengthen the positive) decision-making.

### 3.2. Reasons for Sale or Slaughter of Pregnant Cows

The reasons for sale or slaughter of pregnant cows for meat among herders and abattoir workers in South-Eastern Nigeria are shown in Table 2. Some of the reasons for SPCs and a proportion of the respondents were: ignorance of the animals’ pregnancy status (69.7%, 83/119), high demand for beef (61.3%, 73/148), buyers’ preference for large-sized animals (47.9%, 57/148), feed scarcity during drought (26.9%, 32/148), economic hardship (52.1%, 62/148) and disease conditions (42.9%, 51/148).

### 3.3. Distribution of Pregnant Cows Slaughtered

The age, season and breed distribution of pregnant cows (*n* = 148) slaughtered in South-East Nigeria is shown in Table 3. The spatial and temporal distribution of pregnant cows (*n* = 148) slaughtered in major slaughterhouses in South-East Nigeria is shown in Table 4. The number of cows slaughtered statistically differed across the age groups (*p* = 0.029) and season (*p* = 0.038), but not breed (*p* > 0.05). The majority of the slaughtered pregnant cows (60.1% = prevalence) were in their active reproductive age (4–8 years). Approximately 59% (87/148) of the pregnant animals were slaughtered during the dry/hot season. Most of the pregnant cows (80.4%, 119/148) were of the White Fulani breed. The number of cows slaughtered statistically differed across the month of slaughter (*p* = 0.021), but not slaughter location (*p* > 0.05). Similarly, greater proportions (34.5%) of the pregnant cows were slaughtered in December, while only 38.5% (57/148) were killed at the Akwata slaughterhouse, located in Enugu State, Nigeria.

The age and sex distribution of foetuses (*n* = 148) recovered from pregnant cows slaughtered in South-East Nigeria is shown in Table 5. Furthermore, 55.4% (82/148) of foetuses recovered were male, while 22.3% (33/148), 34.5% (51/148) and 43.2% (64/148) were in their first, second and third trimester of gestation, respectively. Significant association (*p* ≤ 0.05) existed between SPCs and age and season at *p* = 0.029 and 0.038, respectively. In the same vein, there was significant association (*p* ≤ 0.05) between SPCs and months of the year, but none was found between SPCs and slaughterhouse location at *p* = 0.406.

### 3.4. Method of Disposal of Eviscerated Foetuses

The method of disposal of eviscerated foetuses among abattoir workers (*n* = 119) surveyed in South-East Nigeria is shown in Table 6. Eviscerated foetuses or uterine contents were sold for human consumption, 17.6% (21/119); preparation of dog food, 27.7% (33/119) or disposed by open refuse dump methods, 54.6% (65/119). Likewise, 23.5% (28/119) of the slaughterhouse workers surveyed sold foetuses or afterbirth materials for the feeding of pigs or fish (Table 6). Only 5.9% (7/119), and 3.4% (4/119) of the respondents disposed the foetuses or afterbirth materials by burial and incineration, respectively. 

### 3.5. Estimated Economic Losses of SPCs

As regards the economic losses inherent in SPCs and the consequential bovine foetal wastages, if the 148 foetuses were born alive and reared to maturity, this would have yielded 42,890 kg of beef (based on 63% carcass yield and average maturity live weight of 460 kg). If the same numbers of foetuses were wasted in all of the slaughterhouses nationwide, 4.3 tons of beef would have been lost. In monetary terms (based on current market price of ₦ 2000 or $5.3 per kg of beef), this would amount to a loss of ₦ 70.1 million ($186,400), which corresponds to a loss of ₦ 8.6 billion ($23 million) nationwide (data not shown). To estimate the annual economic losses associated in SPCs and the resultant foetal wastages, the aforementioned figures would have to be doubled, since the estimated losses were computed for just a period of six months in which the study lasted.

## 4. Discussion

Identifying the right personnel, particularly with the appropriate experience, as shown in Figure 3, is very crucial in ensuring that the conditions/welfare checks of the cattle and its surroundings are adequately assessed. Additionally, from Figure 2 and Figure 3, the direct observation method is adapted to help ensure that the final decision-making arrived at is evidence-based. Conditions of the lairage, keeping, handling and slaughter conditions are among the key factors deemed needful in ensuring that optimal welfare checks are adequate. On the other hand, Table 1 has elaborated positive and negative conditions foreseeable in various cattle slaughter situations. Particularly, the way-outs further substantiate how to ensure the authenticity, consistency and standardisation of decision-making. Implementing the decision-making strategy herein (refer to Figure 3 and Table 1) can help maintain humane slaughter practices of cattle thriving in slaughterhouses. To see that humane slaughter practices apply to the slaughter animals, some factors have to be considered, for example the number of cattle that: (1) were successfully stunned at the first attempt, (2) were rendered unconscious post first stunning, (3) vocalised during stunning and bleeding, (4) fall during handling, and (5) moved with an electric goad (where applicable) [16,17]. At the post-slaughter of cattle, there could arise carcass/meat defects such as PSE and DFD, which can be associated with an abnormal meat pH. This generally happens when food-producing animals, prior to slaughter, are subject to poor welfare conditions [14]. Such meat defects pose detrimental effects to the quality indices, which impedes profitability and sustainability of any meat production enterprise [15]. Therefore, cruel practices like dragging, dropping, throwing or hoisting during the slaughter process should be prohibited, as it is not part of the humane handling practices [16]. Notably, poor AW together with stress factors can increase disease susceptibility of slaughter-cattle, especially the pregnant ones. Such disease conditions may be zoonotic, and hence transmissible to humans via the food chain [21].

The findings herein, that 17.4% of the cows slaughtered for meat were pregnant, is unacceptably high from AW and animal production perspectives (Table 3). This portends flagrant disregard and insensitivity to the welfare needs of the pregnant cows. To protect the welfare of pregnant animals, the Nigerian Meat Edict of 1968, the Animal Disease (Control) Act of 2004 (as amended), as well as the Council Regulation (No 1/2005) of the EU [57], prohibits the transportation of gravid animals, especially at the late stage of their gestation. The laws/regulations likely foresee that the welfare needs of the dam and that of their foetuses may not be met en route, due to the delicate nature of pregnant animals. Therefore, the SPCs only climaxed the abuse of the animals’ welfare, which was violated *ab initio* when the gravid dams were transported to the abattoirs for slaughter. Although Nigeria does not have a stand-alone legislation on the welfare of animals, legislations guiding the use and ownership of animals, whether in the form of Acts, Code, and Laws, of which some are considered obsolete, have been amended to promote AW. The development of the Nigerian Animal Welfare Strategy Framework in 2016 is one of such amendments. This welfare strategy, which applies to all domestic and captive animals, proscribes all forms of cruelty, deliberate pain or suffering to animals, through negligence or failure to act by the animal owners/custodians/care giver. Additionally, the Animal Disease (Control) Act of 2004 (as amended) provides some additional protections for farm animals, including limiting stocking density during transportation to ensure adequate ventilation. This specific Act also provides for feeding en route, for slaughter/trade animals transported over very long distances. However, there appears to be no significant progress, particularly to ensure the strict implementation of these legal statutes. The operationalisation of standard AW practices in Nigeria remains very challenging, because the nation’s cattle production sub-sector is almost entirely in the hands of “Fulani” herdsmen, who engage in the nomadic system of cattle-rearing that involves (calves or pregnant cows) trekking over very long distances daily in search of food or water [58,59,60].

To actualise the effective implementation of humane slaughter practices in Nigerian slaughterhouses remains very challenging, largely due to the limited mechanised slaughter facilities [61]. Moreover, stunning prior to bleeding during the slaughter process can greatly reduce pain perception. However, the ‘halal’ slaughter widely practiced in Nigeria appears yet to fully embrace stunning, particularly penetrative percussive stunning, even though some workers [62,63] have reported this stunning type able to knock out the consciousness in slaughter animals with ease. Stress due to decreased pre-slaughter welfare conditions (no housing and harsh climatic conditions) observed in this study can lower the immune status of slaughter cattle. This situation can aid the acquisition and dissemination of economically important livestock diseases, thereby worsening the already precarious welfare condition of the animals [64,65,66]. This is where and why the age of the cattle and season of slaughter remains very crucial. In addition, the humane pre-slaughter handling is key to preventing an abrupt increase in the rate of pH decline at the early phases of post-mortem, because this situation can greatly affect both physiochemical and sensory qualities of the meat [7,56,67,68]. For instance, the water-holding capacity (WHC) is a key index of meat that demonstrates the ability of post-mortem muscle to retain moisture despite external pressures (cooking/heating, gravity). Moreover, pre-slaughter stress or exhaustion increases heart and respiratory rates, including muscle metabolic activities. This situation will deplete the muscle glycogen available for enzymatic conversion to lactic acid in the meat tissue, which is required to optimise the post-mortem pH (5.5–5.7) [69,70,71,72], prolong the shelf life, and limit microbial spoilage. Humane pre-slaughter practices would improve the sensory qualities of meat such as tenderness (or texture), juiciness and flavour [73,74].

A number of poor pre-slaughter practices were noted in this study. These included dragging, strangulation and excessive beating, which can increase the levels of creatine kinase (CK) and aspartate aminotransferase (AST) in meat. These enzyme markers are indicative of muscle damage and low meat quality, due to their adverse effects on meat colour (appearance), taste, and other quality indicators [75]. Agbeniga and Webb [76] reported that reducing both anxiety/stress and pre-slaughter stunning enhanced bleed-out, decreased blood retention in the trachea, as well as blood splash in the lungs, which prolonged the meat shelf-life quality and limited the proliferation of spoilage bacteria. Enhanced bleed-out during slaughter is cardinal to food safety, as it limits microbial meat spoilage. Inefficient bleeding during slaughter owing to shock or fright-induced vasoconstriction can withhold blood in edible tissues [77], which facilitates meat decomposition and spoilage bacteria proliferation [78]. Further, high tissue cortisol level caused by pre-slaughter stress also facilitates lipid oxidation and activities of autolysis enzymes, which would enhance putrefaction or formation of PSE meat, even under storage (refrigeration or frozen) conditions [1,12,78,79].

As per the welfare of the foetuses, the scientific debate has been on-going, for and against the bridge of AW inherent in foetal wastages as a result of the SPCs. The contentions focus on whether a foetus can perceive pain during maternal slaughter, and if so, at what stage of gestation does the pain perception begin. While the EFSA [80] affirmed that foetuses do not feel pain during the first and second trimesters, as the relevant physical and neurological structures only develop during the third trimester, there are expert opinions that suggest there could be between a 1%–33% likelihood that foetuses may experience pain during the last stage of gestation (third trimester). Corroborating this, recent expert opinions from the field of developmental neuroscience suggest that pain perception in foetuses may even start earlier than might have been previously thought. Derbyshire and Bockmann [81] reported that the brain cortex and the associated tracts, which are responsible for pain perception, could develop and emerge functional during the latter phase of the second trimester. Despite this, the “foetal pain” may be sub-cortically based, even though the relatively undifferentiated experiences of discomfort may be due to neural processing at levels below the cerebral cortex [82]. For actual pain perception to occur, the foetus needs to be both sentient and conscious [83]. During the gestational life, foetuses are maintained in a sleep-like unconscious mode through the activities of neuro-inhibitors [83]. Consciousness only appears after birth following a substantial withdrawal of the neuro-inhibitors particularly adenosine [83,84]. This unconscious state makes pain perception in the foetus most unlikely. This implies that on event of unforeseen death of the dam, the foetus simply passes from the unconscious state to death with no or minimal pain. This argument, however, does not justify the unapproved slaughter of pregnant animals.

The high rate (17.4%) at which pregnant cows were slaughtered and foetuses recovered (Table 4 and Table 5) shown in this current study is rather surprising. This suggests that AW awareness in the study area appears to be low. With the exception of the SPCs, on the grounds of animal health and other welfare considerations, as may be approved by a veterinarian on ethical grounds, maternal slaughter on the basis of increased meat demand connotes cruelty and is hence unacceptable. It appears that the slaughter of gravid cows and the associated welfare issues is not restricted to Nigeria, as SPCs have been reported in more advanced countries such as the UK [85], Germany [86] and more recently in Denmark [87]. However, the magnitude of the practice and the reasons for the slaughter may vary from place to place. While the EFSA [80] reported that an average of 3% of dairy cows, and 1.5 % of beef cattle slaughtered in the EU were in their last stage of gestation, our findings shows that 43.2% of pregnant cows slaughtered were at their third trimester. The slaughter of terminally gravid cows is worrisome, as pregnancies at the third trimester of gestation should be detected with ease, even by visual assessment. The slaughter of pregnant cows/heifers at the latter stages of pregnancy, as found in this work (Table 5), agrees with the findings of Wosu [40], in which 74% of pregnant cows slaughtered were in their second or third trimesters. This destructive practice does not only indicate animal cruelty but also threatens the development or growth of the livestock industry through excessive off-takes, depletion of the future herd, as well as loss of good genetic traits [88].

In the current study, SPCs within six months would lead to an estimated beef loss of about 44,000 kg, which would be equivalent to about ₦ 70.1 million or $186,400, with consequential foetal wastage. If this were to be replicated nationwide across slaughterhouses, there could be an estimated loss of about 4.3 tons of beef, equivalent to about ₦ 8.6 billion or $23 million. Further, if SPCs are allowed to persist unabated, the already precarious situation of acute animal protein shortfall in the country may worsen. This is because the 21 million cattle in the country, with an average annual growth rate of 1.85% [89], may not provide all the beef and dairy needs of approximately 210 million Nigerians with a population growth rate of 3.5% per annum [26]. Given that most developing countries are yet to achieve self-sufficiency in beef production [90], it is mind blowing to imagine the colossal loss of 4.3 tons of beef or ₦ 8.6 billion ($23 million) due to SPCs and the resultant BFW. These avoidable losses are major setbacks to food security and economic development, in a country where meat supply grossly lags behind the demand [91].

Apart from the losses and the inherent economic wastages, SPCs may facilitate the spread of zoonotic pathogens inhabiting the reproductive tract of cows. *Brucella* and *Leptospira* organisms inhabit the reproductive and urinary tracts. Zoonotic transmission of brucellosis in abattoirs is possible via direct contact with placenta, eviscerated foetuses, uterine secretions or afterbirth materials from infected animals [92,93,94,95]. *Leptospira* infection can result from direct or indirect exposure to body fluids (urine) from the reproductive or urinary tracts of an infected animal [96,97], especially during abattoir meat processing. During the dressing or gutting of an infected animal, the contamination of the processed meat as well as the infection of the slaughterhouse workers would most likely be inevitable, if slaughterhouse workers do not wear personal protective equipment during routine operations [98]. It is also possible for the abattoir environment to be contaminated with the zoonotic organisms from food animals grazing around infected areas, which become onward transmissions of the pathogens to humans. This can apply to eviscerated foetuses discarded by unconventional methods (Table 6), which can aid environmental contamination and disease spread.

In all ramifications, the conduct of SPCs for meat would not be profitable. Herders, who may have erroneously culled their cows for infertility reasons, probably due to poor proficiency in pregnancy detection, would be at a loss. The butchers and beef sellers are equally at loss, as pregnant animals, especially those in their late stage of pregnancy, yield less meat than non-pregnant ones [87,99,100]. Further, the quality of meat from pregnant animals is doubtful. Meats sourced from pregnant animals are watery, have high pH and peak shear force values, poor eye appeal and also smell and tastes abnormal, due to high progesterone tissue levels [100,101]. In order to remedy the problem of slaughtering pregnant cows, decisive steps should be taken to provide trained personnel who are proficient in pregnancy diagnosis at the slaughterhouses. One major step in this regard is the engagement of more veterinarians and other trained animal health workers as meat inspectors and animal attendants, respectively. In Nigeria, the local and state governments have the responsibility to oversee and regulate the operations of slaughterhouses domiciled in their locality; however, some governments have contracted the oversight and regulatory functions to privately owned organisations. As a result, ante-mortem inspections, including pregnancy diagnosis in female animals intended for slaughter, have been reduced to mere collection of revenue per animal slaughtered [102,103]. To worsen the problem, veterinarians, who by training are competent on pregnancy and disease diagnosis in animals, are hardly employed by the contractors, probably to save costs. This development may have resulted in the high rate of SPCs being reported, and should be reversed by engagement of trained personnel at the abattoirs to improve the welfare conditions of slaughter-cattle and halt the SPCs.

Seasonality in the SPCs demonstrated herein (Table 3) could be a useful clue exploitable for effective control of AW challenges during the dry/hot seasons. A huge quantity of animal feed could be stocked and sold off to livestock farmers during draught and/or festive periods, at a subsidised rate, as an incentive to prevent sale or slaughter of gravid cows for meat. Significant loss of body conditions, as a result of drought-related food and water scarcities during the dry/hot season, usually compels livestock owners to salvage their animals by selling them off, irrespective of their pregnancy status [89,104]. Additionally, diversification of meat production through intensification of poultry production and other food animals could lessen the increased meat demand during festive periods, which usually prompts livestock farmers to sell off their animals, including pregnant ones, as buyers tend to offer higher prices. On the part of livestock farmers, there is a need for supervised breeding (especially in outdoor/extensive farming systems), proper recording of all breeding activities and routine on-farm pregnancy testing. This will help to limit farmers’ ignorance on the pregnancy status of their animals, which was one of the major factors underpinning the sale of SPCs for meat (Table 2). In this regard, farmers’ training and retraining of abattoir workers on some on-farm and rapid pregnancy diagnostic methods will be a step in the right direction. Also, massive public enlightenment campaigns on AW and the need to curtail the SPCs for meat is recommended, since the welfare of animals, particularly slaughter-cattle, is not prioritised in most developing countries.

## 5. Concluding Remarks

The slaughter of pregnant cows (SPCs) and its implications on the meat quality and food safety/security has been examined in this current study. Of the 851 cows, 17.4% (148/851) were pregnant while 43.2% (64/148) of the total foetuses recovered were in their third trimester. The finding that 17.4% of the cows slaughtered for meat were pregnant is unacceptably high from AW and animal production perspectives. The high rate (17.4%) at which pregnant cows was slaughtered is rather surprising, and suggests that AW awareness in the study area appears low. In this study, SPCs within six months would estimate beef loss amounting to 44,000 kg, which would be equivalent to ₦ 70.1 million or $186,400, with consequential foetal wastages. Despite this, the SPCs for meat is unprofitable in all ramifications, and its seasonality demonstrated herein could be useful clue for effective control of AW challenges in slaughterhouses in South-East Nigeria.

Strict implementation of the provisions of the Meat Edict of 1968 and Animal Disease (Control) Act of 2004, including humane slaughter and the prohibition of slaughter of PFAs, remains imperative. In fact, an amendment of the laws or enactment of the Nigerian Animal Welfare Strategy Framework drafted in 2016 in line with the present day AW issues, particularly at the slaughterhouses, is long overdue. Incorporating compulsory pregnancy diagnosis of all slaughter female animals in the ante-mortem inspection protocol is warranted. Proper labelling of meats obtained from pregnant animals, to guide buyers’ discretion, should be mandatory. Slaughter facilities and persons defaulting on the issue of humane slaughter practices should be publicly reprimanded to deter others from doing the same. Overall, SPCs for meat and inhumane slaughter practices at the slaughterhouse should be prevented, to enhance the welfare needs of slaughter cattle, grow the national herd size, and improve meat safety as well as food security in Nigeria.

## Figures and Tables

**Table 1:** Positive and negative cattle welfare conditions and corresponding way-outs, from environment conditions at the lairage, up to the actual slaughter process/practices.

Positive Cattle Welfare Condition	Negative Cattle Welfare Condition	Way-Outs/Remarks
CATTLE ENVIRONMENT AT THE LAIRAGE		
* Adequate shelter at the lairage	* No shelter, marshy or water-logged floor	Provision of shelter, adequate floor space, feed/water and conducive environment at the holding pen/lairage; to limit overcrowding, stress, fighting and hence injuries and lacerations
** Moderate stocking density and hence adequate floor space	** Overcrowded or high stocking density	
** Absence of wounds, lacerations or fight injury	** Presence of wounds, lacerations and injuries/fractures due to fighting caused by overstocking or starvation/thirst	
** Ability to express innate abilities like mooing, jumping, etc	** Depressed, afraid, in a state of pain and hence unable to express innate abilities	
PRE-SLAUGHTER CATTLE CONDITION		
** No slipping or falling ** No hitting or mal-handling ** No dragging to the kill floor ** No vocalisation/groaning during handling	** Dragging, hitting, falling, groaning, etc during pre-slaughter handling	Training and retraining on pre-slaughter human handling. Mechanisation of slaughter and processing processed to limit excessive use of force or manual labour. Enactment and or strict implementation of human animal handling before and during slaughter.
DURING SLAUGHTER OF CATTLE		
** Stunning prior to bleed	** No stunning prior to bleeding	Training and retraining on modern and human slaughter practices. Mechanisation of slaughter and processing processed to limit excessive use of force or manual labour. Provision of modern slaughter facilities to automate slaughter process and limit meat contamination. Enactment and or strict implementation of human animal handling before and during slaughter.
** Stunning to bleeding interval of less than 5 s	** Stunning to bleeding interval of ≥ 5 s	
** No dropping, throwing or hoisting during the slaughter	** Dropping, throwing or hoisting during the slaughter	
** All animals successfully stunned (were rendered unconscious) at the first attempt.	** No or few animals successfully stunned (were rendered unconscious) at the first attempt	
** No vocalisation/groaning during stunning or bleeding	** Vocalisation/groaning during stunning or bleeding	

* Veterinarians’ field experience; ** Sourced from published works [2,16,54,55,56].

**Table 2:** Reasons for sale or slaughter of pregnant cows for meat among herders and abattoir workers (*n* = 119 *) in South-Eastern Nigeria.

Reasons/Causes	Number (%) of Respondents		
	Yes	No	No Response
High demand for beef	73 (61.3)	21 (17.6)	25 (21.0)
Economic hardship	62 (52.1)	57 (47.9)	0
Ignorance of the pregnancy status of the animal	83 (69.7)	32 (26.9)	4 (3.4)
Preference for pregnant cows because of size	57 (47.9)	46 (38.7)	16 (13.4)
Feed scarcity during dry seasons	32 (26.9)	87 (73.1)	0
Disease conditions	51 (42.9)	42 (35.3)	26 (21.8)

* Total number of herders and abattoir workers in the 3 slaughterhouses surveyed.

**Table 3:** Age, season and breed distribution of pregnant cows (*n* = 148) slaughtered in South-East Nigeria.

Variables	Number (%) of Cows Slaughtered	Number (%) of Pregnant Cows Slaughtered	Prevalence	*p*-Value
*AGE*				
<4 years	126 (14.8)	30 (23.8)	20.3	0.029 *
4–8 years	498 (58.5)	89 (17.8)	60.1	
>8 years	227 (26.7)	29 (12.8)	19.6	
SEASON				
Wet	418 (49.1)	61 (14.6)	41.2	0.038 *
Dry	433 (50.9)	87 (20.1)	58.8	
BREED				
White Fulani	621 (72.9)	119 (19.2)	80.4	0.149
Sokoto gudali	130 (15.3)	18 (13.8)	12.2	
Red bororo	63 (7.4)	7 (11.1)	4.7	
Mixed breeds	37 (4.3)	4 (10.8)	2.7	

* Significant statistical association (*p* ≤ 0.05), Fisher’s exact test.

**Table 4:** Spatial and temporal distribution of pregnant cows (*n* = 148) slaughtered in major slaughterhouses in South-East Nigeria.

Variables	Number (%) of Cows Slaughtered	Number (%) of Pregnant Cows Slaughtered	Prevalence	*p*-Values
MONTHS				
December	197 (23.1)	51 (25.9)	34.5	0.021 *
January	157 (18.4)	24 (15.3)	16.2	
February	129 (15.2)	21 (16.3)	14.2	
July	118 (13.9)	17 (14.4)	11.5	
August	121 (14.2)	16 (13.2)	10.8	
September	129 (15.2)	19 (14.7)	12.8	
SLAUGHTER LOCATIONS				
Enugu	327 (38.4)	57 (17.4)	38.5	0.406
Nsukka	315 (37.0)	49 (15.6)	33.1	
Awka	209 (24.6)	42 (20.1)	28.4	

* Significant statistical association (*p* ≤ 0.05), Fisher’s exact test.

**Table 5:** Age and sex distribution of foetuses (*n* = 148) recovered from pregnant cows slaughtered in Southeast, Nigeria.

Variables	Number (%) of Foetuses
AGE	
First trimester	33 (22.3)
Second trimester	51 (34.5)
Third trimester	64 (43.2)
SEX	
Male	82 (55.4)
Female	66 (44.5)

**Table 6:** Method of disposal of eviscerated foetuses among abattoir workers (*n* = 119) surveyed in South-East Nigeria.

Method of Foetus Disposal *	Number (%) of YES Respondents
Sold foetuses for human consumption	21 (17.6)
Sold foetuses for preparation of dog food	33 (27.7)
Sold eviscerated foetus for feeding of fishes or pigs	28 (23.5)
Dumped unsold foetus or gravid uterine contents in municipal refuse dump	65 (54.6)
Incinerated unsold foetus or gravid uterine tissues	4 (3.4)
Buried unsold foetus or gravid uterine contents	7 (5.9)

* Many respondents disposed foetuses by more than one method.

## Data Availability

Data sharing not applicable.
